# Abiotic Stresses in Plants: From Molecules to Environment

**DOI:** 10.3390/ijms25158072

**Published:** 2024-07-24

**Authors:** Martin Bartas

**Affiliations:** Department of Biology and Ecology, Faculty of Science, University of Ostrava, 710 00 Ostrava, Czech Republic; martin.bartas@osu.cz

## 1. Foreword

Plants face several challenges during their growth and development, including environmental factors (mainly abiotic ones), that can lead to/induce oxidative stress—specifically, adverse temperatures (both hot and cold), drought, salinity, radiation, nutrient deficiency (or excess), toxic metals, waterlogging, air pollution, and mechanical stimuli. With the increasing frequency of extreme weather events due to ongoing climate change, understanding how plants sense and adapt to these stresses will become more and more crucial for ensuring food security and sustainable agriculture. In this Special Issue, a diverse array of original articles was released, encompassing multidisciplinary approaches and methods of bioinformatics, in vitro and in vivo experiments, and innovative field trials, some of which are poised for practical implementation. Additionally, a collection of comprehensive reviews dedicated to various aspects of the issue was also published. This editorial aims to provide a concise entry point into the problems in this area and introduce the reader to the newest advances in this exciting field, where basic scientific discoveries meet future applications.

## 2. Abiotic Stresses in Plants

**Figure 1:**
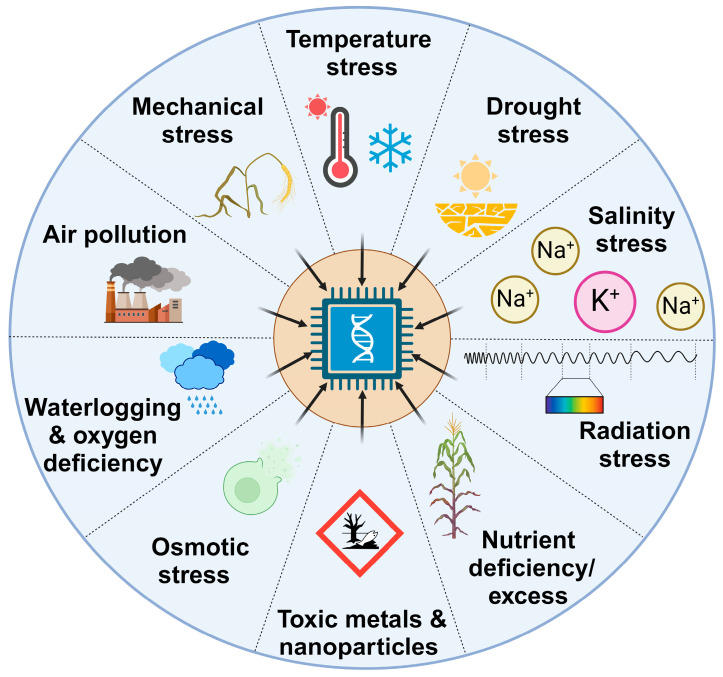
Abiotic stresses in plants. Diagram of most important abiotic stresses in plants (outer circle) which, sooner or later, elicit an integrative response, often involving complex signaling pathways and proteins, interacting with DNA, and inducing the expression of target “stress-responsive genes” (inner circle). This figure was created in BioRender.

All living organisms must cope with various stresses during their lifecycle. In contrast to most animals, plants must withstand adverse conditions in the place where they were “born”; they cannot quickly escape from sun to shadow, from nutrient-poor spot to nutrient-rich venue, or from flooded space to safety. Therefore, during evolution, plants acquired what is likely one of the most sophisticated mechanisms for dealing with a variety of abiotic stresses [1], which are schematically depicted in Figure 1. It is worth mentioning that an extreme condition for one plant species might be an optimal condition for another.

Plants exhibit an ability to adapt to a wide range of temperatures by reprogramming their transcriptome, proteome, and metabolome [2,3]. *Temperature stress* covers both cold and heat stress; interestingly, the involved molecular pathways are distinct to some extent, leading to the expression of either cold-responsive or heat-responsive genes [2].

*Drought stress* in plants significantly disrupts the balance between water uptake and loss. Subsequent physiological and molecular responses aim to conserve water and tolerate stress via stomatal closure, reduced cell expansion, altered photosynthetic activity, or synthesis of osmoprotectants [4] to mitigate the damaging effects of drought stress and enhance plants’ survival in water-deficient environments.

*Salinity stress* in plants arises from excessive salt accumulation in the soil. When exposed to salinity, plants activate homeostatic mechanisms to counter the imbalance caused by salt. This activation involves triggering various signaling components, including the Salt Overly Sensitive (SOS) pathway, the abscisic acid (ABA) pathway, reactive oxygen species (ROS), and osmotic stress signaling, to mitigate the effects of salinity [5].

*Radiation stress* in plants usually refers to exposure to various excessive sources of radiation (including gamma rays and ultraviolet (UV) radiation) that can induce DNA damage, hinder photosynthesis, and impair overall plant growth and development [6]. Photoreceptor protein UVR8 is considered a main sensing component for both UV-B and UV-A [7]. Subsequently, plants respond to radiation stress by activating various protective mechanisms, including the synthesis of UV-absorbing compounds and the enhancement of antioxidant defense systems [8]. In addition, the spectral quality and quantity of visible light affect plant growth and stress reactions [9,10].

*Nutrient deficiency stress* refers to plants lacking a sufficient quantity and quality (e.g., redox state and form of compound/salt) of essential nutrients required for their growth and development. Plants respond to this through a range of adaptive mechanisms, including altered root architecture [11], increased nutrient uptake efficiency, and the reprogramming of metabolic pathways [12]. Additionally, nutrient deficiency stress can impact the synthesis of important biomolecules, such as chlorophyll and proteins, further influencing plant growth. Generally, nutrients in plants exist in three states: deficiency, optimality, and excess [13]. The latter can be damaging not only to the plant but also to the local soil microenvironment and plant symbionts.

*Stress induced by toxic metals* in plants occurs when exposure to excessive levels of metals such as cadmium, lead, or arsenic disrupts essential physiological processes, leading to oxidative stress, growth inhibition, and cellular damage [14]. Plants respond to this by activating detoxification mechanisms, sequestering toxic metals in vacuoles, and producing chelating compounds [15]. *Nanoparticles* are also capable of inducing oxidative stress and adverse effects in plants [16]; however, some of them can also have a beneficial effect on plant growth, such as in cross-tolerance (when plants are exposed to a particular type of stress, it can trigger shared signals and pathways, leading to increased resilience against diverse forms of stress [17]) and related mitigation of stress induced by high salinity in the environment [18].

*Osmotic stress* occurs when plants are subjected to conditions that disrupt the balance of water and solutes within their cells, leading to significant changes in water potential (ψw) [19]. It can occur at the initial stage of plant exposure to other abiotic stresses and induces specific kinases, such as the SNF1-related protein kinase (SnRK) 2 family, which is essential for osmotic stress signaling [20]. Osmotic stress often leads to physiological damage, impairs ABA-mediated stomatal closing, and decreases survival in drought and salinity stresses [20].

The main cause of *oxygen deficiency stress* in plants is environmental factors such as excessive rainfall leading to soil waterlogging or complete submergence of plants, which can result in a shortage of oxygen (hypoxia) or a total absence of oxygen (anoxia) [21,22]. Under these conditions, a series of rapid molecular and metabolic responses are activated to endure such stress, including the induction of specific kinases and the activation of various cellular signaling networks to cope with the low-oxygen environment [23].

*Air pollution stress* (represented mainly by ozone, nitrogen oxides, and suspended particulate pollutants) increases the production of reactive oxygen species (ROS) in plants, leading to oxidative stress and potential damage at various levels (including the disruption of essential functions like transpiration, reduced resistance to heat stress, nutrient deficiencies, and subsequent illnesses) [24]. Furthermore, air pollution can shift the competitive balance among various plant species and may lead to changes in the composition of the plant community, altering the structure and function of the ecosystem [25].

Last but not least, *mechanical stress* encompasses a range of physical forces such as wind, touch, heavy rain, or vibrations, which can significantly influence plant growth and development [26]. These stresses trigger a variety of responses, including changes in gene expression, hormone signaling, and cell wall composition, to help the plant adapt and withstand these mechanical forces. Additionally, mechanical stress can lead to alterations in plant architecture, including stem thickening, root system modification, and changes in leaf orientation, as the plant adapts to its environment and attempts to minimize damage from external forces [27]. Interestingly, herbivores can also induce significant mechanical stress in plants, e.g., through feeding or trampling, prompting the production of “bitter” secondary metabolites that serve as a defense mechanism against further herbivory [28].

It is worth mentioning that not all stresses are equal. Wild plants in their natural habitats are usually *adapted* to the specific extreme conditions of a particular environment and have developed very effective protective mechanisms. On the other hand, cultural plants/crops often have to deal with severe abiotic stresses which are partly a consequence of unsustainable agriculture and accompanying phenomena, such as soil erosion and loss of groundwater [29,30].

From a basic research perspective, there is still a limited understanding of the combined effects of two or more abiotic stresses on plant physiology and development, as well as their synergistic or antagonistic impacts [31,32]. Moreover, the combination of abiotic and biotic stresses can lead to even more intricate interactions, affecting the plant’s molecular and metabolic responses [33].

Abiotic stresses in plants represent one of the main challenges in current agriculture. Due to ongoing climate change, there is a need for a deeper understanding of how plants deal with various environmental cues, especially on a molecular level. The recent integration of multidisciplinary approaches, such as omics technologies, bioinformatics, and computational modeling, is facilitating a comprehensive understanding of the complex interactions between plants and their environment [34], pointing to the original name of this Special Issue, “Abiotic Stresses in Plants: From Molecules to Environment”.

## 3. Short Overview and Summary of Published Articles

Let us delve into specific articles featured in this Special Issue and highlight their key focus and main message. I encourage readers to peruse this list and explore the full papers that pique their interest.

A short communication by Bartosz J. Płachno et al. highlights the effectiveness of the continuous and well-developed cuticle in *Drosophyllum lusitanicum* for water conservation in arid environments and provides insights into the challenges and techniques associated with studying the composition of cell walls in glands of this carnivorous plant.

Yucun Yang et al. developed a rapid and nondestructive method based on hyperspectral imaging for the evaluation of wheat (*Triticum aestivum* L.) chlorophyll under drought stress, which may be particularly useful for high-throughput phenotypic analysis and the genetic breeding of crops in general.

The article by Hongying Chen, Anne M. Visscher et al. investigates the intra-speci variation in desiccation tolerance of *Citrus sinensis* ‘*bingtangcheng*’ (L.) seeds, highlighting the influence of maternal environmental conditions, such as annual sunshine hours and temperature, on seed survival during dehydration, as well as the stable expression levels of stress-responsive genes in modulating desiccation tolerance.

Research conducted by Lixian Wei, Xin Zhao et al. identified 17 Caffeoyl-coenzyme A-O-methyltransferase encoding genes (*CCoAOMTs*) in *Dendrocalamus farinosus*, revealing that they are significantly associated with lignin content, xylem thickness, and drought resistance in transgenic plants. This posits them as potential candidate genes involved in the drought response and lignin synthesis pathway in plants, with potential implications for the genetic improvement of important traits in *Dendrocalamus farinosus* and other species.

A study by Jinghan Peng, Siyu Liu et al., based mostly on bioinformatics, facilitated a comprehensive identification of twenty *HSP90* genes in oats (*Avena sativa*), elucidating their evolutionary pathways and responses to various abiotic stresses and demonstrating the significant up-regulation of specific *HSP90* genes under heat stress.

Dariel López et al. demonstrate that nocturnal warming has a more pronounced impact than diurnal warming on the cold deacclimation process in *Deschampsia antarctica*, influencing the expression of freezing tolerance-related genes and the plant’s response to cold stress. These findings provide valuable insights into the effects of asymmetric warming on plants’ adaptive responses and survival in a changing environment.

The research by Mason T. MacDonald et al. uncovers the relationship between lipid changes during cold acclimation and postharvest needle abscission in balsam fir (*Abies balsamea*), highlighting the practical significance of identifying peak needle retention and suggesting the potential use of the monogalactosyldiacylglycerol (MGDG) to digalactosyldiacylglycerol (DGDG) ratio as a screening tool for balsam fir genotypes with stronger needle retention characteristics.

The genome-wide analysis conducted by Jiafang Shen et al. identified seven genes encoding sucrose phosphate synthase (*SPS*) in soybean (*Glycine max*). The authors then investigated their tissue expression patterns and cold stress response. The study highlights the potential roles of *SPS* genes in soybean’s response to cold stress and their implications for future research in this area.

Valentin Ambroise et al. investigated the impact of long-term mild metal exposure on the cold acclimation of *Salix viminalis* roots and revealed that simultaneous exposure to both stressors results in unique effects. The study also highlights the under-studied role of lignans and the ROS damage repair and removal system in plants facing multiple stressors.

The study by Xuan Ma, Qiang Zhang et al. identified the molecular basis of salt resistance in two poplar species (*Populus alba* and *Populus russkii*), revealing that *Populus alba* exhibited enhanced energy metabolism, superior Na^+^ transportation, different regulation of stress-related genes, and an overall improved ability to thrive under salt stress.

Yong Chen, Wanling Yang et al. focused their efforts on the miRNA-mediated salt stress response in Dongxiang wild rice (*Oryza rufipogon* Griff.), identifying 874 known and 476 novel miRNAs. This sheds light on the regulatory mechanisms of salt stress tolerance in this wild rice, offering insights that could potentially improve salt tolerance in cultivated rice in the future.

The study by Ziwei Li et al. sheds light on the regulation mechanism of a polymer soil amendment (PPM) on rapeseed (*Brassica napus* L.) photosynthesis under drip irrigation with brackish water. The research highlights the differential responses of rapeseed phenotype, photosynthetic physiology, transcriptomics, and metabolomics to PPM application with different types of brackish water, providing insights for addressing salinity stress in crops caused by drip irrigation with brackish water.

The study by Mingjie Ren, Jingjing Ma et al. unveils the molecular mechanisms behind Magnolia’s (*Magnolia sinostellata*) sensitivity to shade, highlighting the critical role of the *STAY-GREEN* gene in regulating chlorophyll degradation under light deficiency stress.

Miho Ohnishi, Shu Maekawa et al. author two articles in this issue. The first investigates the activity of ferredoxin (Fd)-dependent cyclic electron flow (Fd-CEF) around photosystem I (PSI) in intact leaves of *Arabidopsis thaliana*, highlighting the relationship between the oxidation rate of Fd reduced by PSI (vFd) and photosynthetic linear electron flow activity and discussing the physiological significance of the excessive vFd observed under higher photosynthesis conditions. The second (follow-up) study is devoted to the enhanced reduction of Fd in the PGR5-deficient mutant of *Arabidopsis thaliana,* which is stimulated by Fd-CEF around PSI. The authors found that the protein PROTON GRADIENT REGULATION 5 (PGR5) is probably not catalyze Fd-CEF, which challenges previous assumptions about its role in this process.

The study by Karolina Stałanowska et al. reveals the size-dependent harmful effects of zinc oxide (ZnO) nanoparticles on the germination and seedling growth of garden pea (*Pisum sativum* L.) and wheat (*Triticum aestivum* L.), emphasizing the potential phytotoxic risks associated with soil contamination by ZnO nanoparticles.

The study by Sandra S. Scholz et al. demonstrates that the root-colonizing endophytic fungus *Piriformospora indica* facilitates the metabolomic adaptation of its host plant (*Arabidopsis thaliana*) against nitrogen limitation by delivering reduced nitrogen metabolites, thereby alleviating nitrogen starvation responses and reprogramming the expression of nitrogen metabolism-related genes.

Vida Nasrollahi et al. investigated the role of Squamosa-Promoter Binding Protein-Like 9 (SPL9) in nodulation in the important crop alfalfa (*Medicago sativa*), revealing that MsSPL9 regulates nodulation under high-nitrate conditions by modulating the expression of nitrate-responsive genes.

The study by Ying Guo, Yongli Qi et al. focuses on grafted ginkgo trees (*Ginkgo biloba* L.) in different regions of China and involves RNA-seq, small RNA-seq, and metabolomics data to elucidate the regulatory mechanism of terpene trilactone (TTL) biosynthesis in ginkgo leaves. The authors identified differentially expressed miRNAs and transcription factors that play a role in the environmental response and provide insights for improving the medicinal value of ginkgo leaves under global climate change.

The research by Xi-Min Zhang et al., which focused on *Rhododendron delavayi* under waterlogging stress, revealed significant physiological and molecular changes, including significantly reduced CO_2_ assimilation and chlorophyll content, excessive H_2_O_2_ accumulation, and altered metabolic pathways.

The review by Bowen Tan and Sixue Chen focuses on the mechanisms of the C3-to-CAM photosynthesis transition, an adaptive form of photosynthesis in hot and arid regions, which provides promising solutions for creating stress-resilient crops in the face of global climate challenges.

The review by Shuya Tan, Yueqi Sha et al. provides an overview of the molecular mechanisms triggering leaf senescence under abiotic stresses, emphasizing the significance of understanding these processes to enhance crop resilience and productivity in unfavorable environments.

The review by Li Yang et al. highlights the impact of salinity on cotton growth and fiber yield, focusing on the roles of S-adenosylmethionine in ethylene (ET) and polyamine (PA) biosynthesis and signal transduction pathways. Improved regulatory pathways of ET and PAs under salt stress in cotton could be potentially used for the breeding of salt-tolerant varieties.

The last published review is authored by Adriana Volná et al. and provides insights into the regulation of genes related to phenolic compound production concerning the full route from photoperception to transcription control and gene expression. Such knowledge enhances our understanding of plant responses to UV and visible light and offers potential avenues for manipulating the content and profile of phenolic compounds, with implications for horticulture and food production.

## 4. Conclusions

In this Special Issue, a total of 25 articles were published, completely outperforming my modest expectations (List of Contributions). Out of this number, there were 20 original research articles, 4 comprehensive reviews, and this editorial, which is slowly concluding. I hope that the readers of this Special Issue find the articles published herein useful and inspirational; the field of *Abiotic stresses in plants* is highly dynamic and rapidly evolving, which provides many opportunities for young scientists to find their research niche.
